# A Biopsychosocial and Environmental Perspective of Youth Health Literacy in Portugal

**DOI:** 10.3390/children12040410

**Published:** 2025-03-24

**Authors:** Tania Gaspar, Miguel Arriaga, Marina Carvalho, Fábio Botelho-Guedes, Ana Cerqueira, Margarida Gaspar-Matos

**Affiliations:** 1 Digital Human-Environment Interaction Labs (HEI-LAB), Lusófona University, Campo Grande, 1749-024 Lisbon, Portugal; 2 Institute of Environmental Health (ISAMB), 1649-026 Lisbon, Portugal; marina.carvalho@ismat.pt (M.C.); fabioguedes@edu.ulisboa.pt (F.B.-G.); ana.cerqueira@edu.ulisboa.pt (A.C.); mmmatos@ucp.pt (M.G.-M.); 3 Aventura Social, 1649-026 Lisbon, Portugal; 4 Faculty of Medicine, University of Lisbon (FMUL), Campo Grande, 1649-028 Lisbon, Portugal; 5 Direção Geral da Saúde, 1000-123 Lisbon, Portugal; miguelarriaga@dgs.min-saude.pt; 6 Manuel Teixeira Gomes Higher Institute, University Hospital Centre of Algarve, 8000-386 Portimão, Portugal; 7 Instituto Superior Manuel Teixeira Gomes, 8500-590 Portimão, Portugal

**Keywords:** health literacy, adolescents, ecological approach, mental health, environmental health

## Abstract

Background: From a biopsychosocial perspective, health literacy is a key factor for healthy development and the development of more comprehensive interventions directed at health literacy determinants. The present study had the main goal of studying demographic, individual, social, and contextual variables related to health literacy in adolescents. Methods: The data used in this study are part of the Health Behavior in School-aged Children (HBSC) 2022 survey. The study included 7649 adolescents, 53.9% (*n* = 3961) female, with an average age of 15.05 years (*SD* = 2.36), in the 6th, 8th, 10th, and 12th grades, proportionally distributed across the five regions of the Portuguese mainland. Results: Health literacy was explained by factors related to physical, psychological, social, and environmental health. The factors with the higher explanatory value were the psychological variables, followed by social and lifestyle-related variables. Sociodemographic and environmental factors had a more modest explanatory value. These results point to the complexity of adolescents’ health literacy. Conclusions: These results are of the utmost importance for educators, professionals, and policymakers who can use this information to create friendly environments that promote health literacy and health-promoting activities according to a multidisciplinary, continuous, and consistent plan.

## 1. Introduction

Health literacy, defined as both the cognitive and social skills underlying the motivation and ability of individuals to access, understand, and use information for promoting and maintaining good health [1,2,3,4], is a life-long learning process starting in early childhood [5]. From a biopsychosocial perspective, health literacy is a key factor for healthy development. Thus, understanding the role of health literacy in a health-promoting lifestyle profile and health status may contribute to a more comprehensive assessment and the implementation of targeted interventions directed to health literacy determinants.

Childhood and adolescence are core stages in which developmental (physical, individual, social, and contextual) processes take place and are associated with individual differences in health-promoting behaviors and lifestyle habits, which, in turn, may later affect adolescents’ quality of life in adulthood [6,7]. Therefore, the early communication and understanding of health information require careful consideration due to its role in enhancing health literacy in adolescents, favoring more positive outcomes in adulthood.

Although there are several studies focusing on the effects of poor health literacy on adult health, studies involving adolescents are still scarce. Considering that adolescence is a window of opportunity for improving health literacy, the analysis of the biopsychosocial and contextual determinants of health literacy is most important [6]. A literature review carried out with the main purpose of understanding the definitions of health literacy and the antecedents and consequences of health literacy in children and adolescents allowed the identification of several individual (e.g., expectations, communication, self-efficacy), demographic (e.g., age, SES), situational, and contextual (e.g., parental health status and health behaviors, support from peers, families, and schools) factors as antecedents of health literacy. On the other hand, the authors [6,7] identified that health literacy in children and adolescents leads to benefits at individual (more skills to make sound decisions and establish and maintain health-related goals), community (extended to a range of life’s activities), and societal (by increasing community participation and empowerment and the capacity to influence one’s and others health) levels. In another systematic review of the studies carried out about the relationship between health literacy and health behaviors in adolescents [8], a significant relationship between health literacy and adolescents’ health behaviors was also found, despite the limitations of the analyzed studies, namely the conceptualization and operationalization of health literacy. Another literature review [9] studied specifically the role of digital health literacy in children and adolescents. The results showed the relevance of health literacy for optimal physical, emotional, and cognitive development and for the promotion of healthy behaviors and lifestyles. In this literature review, the level of health literacy depended on health and socioeconomic status and social and demographic health determinants, among other variables, showing that health literacy needs to be understood in the context in which children live and develop.

When we consider adolescents’ psychological health, the literature evidences that mental health literacy is related to better mental health outcomes (e.g., [10]). Some studies defend that mental health literacy may present a protective role in the relationship between stress management and mental health outcomes (e.g., [11]) and on well-being [12], particularly in girls [13].

In a cross-sectional study developed to study adolescent health literacy, health-promoting lifestyle profile, health status, and related factors [14], the authors found that health literacy and health-promoting lifestyle profiles significantly correlated in the expected directions with perceived health status and depression. In this study, regular exercise was a predictor of health literacy, a healthy lifestyle profile (including interpersonal support), and better psychological health status.

Also, health literacy has been shown to be related to exercise participation [13], the intake of vegetables and fruit [15], vaccines and vaccination [16], sleep (e.g., [17]), substance consumption (e.g., [18,19]), screen time [20], and body image [21] seem to moderate the relationship between bullying and anxiety and depression symptoms [22].

In summary, the reviewed studies about health literacy focused on social, family, school, and individual aspects or on the adolescent’s health condition. However, a biopsychosocial, environmental, and comprehensive view of adolescent health literacy, identifying and integrating variables from the different levels of the ecological model, was not identified in the literature.

Considering the need to understand, from a biopsychosocial perspective, health literacy determinants, the present study’s main goal is to study demographic, individual, social, and contextual variables related to health literacy in adolescents.

## 2. Materials and Methods

The data used in this study are part of the Health Behavior in School-aged Children (HBSC) 2022 survey [23,24]. This survey is carried out every 4 years in collaboration with the World Health Organization (WHO) and follows a strict international protocol [25].

The HBSC/WHO aims to study adolescents’ health behaviors and habits in their life contexts and their influence on their health/well-being. In Portugal, the HBSC/OMS 2022 study has the approval of the Ethics Committee of the Academic Center of Medicine of Lisbon of the Lisbon North Hospital Center and the General Directorate of Statistics for Education and Science (DGEEC). In this edition, 40 Portuguese primary and secondary education school groups participated, totaling 452 classes. The school groups participated voluntarily, and informed consent was obtained from all participants’ parents or legal guardians. Responses to the questionnaire were obtained online and anonymously. The sample is representative of the grades under study. More details about the data collection procedures for the HBSC/WHO 2022 study in Portugal can be found in [23].

### 2.1. Participants

The study included 7649 adolescents, of which 53.9% (*n* = 3961) were female, with an average age of 15.05 years (*SD* = 2.36). The sample included students in the 6th, 8th, 10th, and 12th grades, proportionally distributed across the five regions of the Portuguese mainland (North, Center, Lisbon, Tagus Valley, Alentejo, and Algarve).

Table 1 presents the demographic characteristics of the study participants.

### 2.2. Measures and Variables

Table 2 shows the variables considered in this study, including sociodemographic variables, psychological variables, lifestyle variables, social and relational variables and, finally, contextual and environmental variables.

### 2.3. Data Analysis

Data were analyzed using SPSS (Statistical Package for Social Sciences), version 27 for IOS. An analysis of descriptive statistics was carried out to characterize the sample.

Linear regressions, using the stepwise method, were used to analyze the explanatory weight of each independent variable on the dependent variable, health literacy. Before carrying out the linear regression models, a correlation analysis was conducted between the dependent variable (health literacy) and the other variables, all of which proved to be statistically significant. A significance level of *p* < 0.05 was used.

## 3. Results

Linear regression models were performed to understand adolescents’ health literacy from a biopsychosocial and environmental perspective, first from a global ecological analysis and then from a dimension-by-dimension analysis.

The model presented in Table 3 aims to understand health literacy from a biopsychosocial and environmental perspective. The global model included all the variables under study (i.e., sociodemographic, psychological, lifestyle, social, contextual, and environmental) and was statistically significant, *F* (26; 5609) = 61.121; *p* ≤ 0.001, explaining 22% of the variance.

The results evidenced that several of the studied factors contributed to explaining adolescents’ health literacy. Thus, gender, age, well-being, future expectations, body image, physical activity in the last 7 days, vegetable intake, having up-to-date vaccinations, sleep hours, violence (aggressor and victim), family support, online time with friends, and school pressure were predictors of health literacy. On the other hand, stress, screen time, drug use, relationships with teachers and peers, the use of social media, and safety at school negatively predicted health literacy. Therefore, higher well-being, less stress, more positive future expectations and body image, practicing more physical activity, eating vegetables, having up-to-date vaccinations and healthy sleep habits (sleeping 9 h or more), spending less time exposed to screens, not having consumed drugs or engaged in violent behavior (as aggressor and victim), better family support, relationships with teachers and classmates, more online contact with friends, less use of social networks, feeling more school pressure, and more safety at school were factors associated with higher health literacy in adolescents.

Table 4 presents the health literacy model with sociodemographic variables (i.e., gender, age, and SES). The model was significant *F* (3; 5632) = 21.984; *p* ≤ 0.001 and explained 1% of health literacy. Of all the included variables, only SES contributed to explaining health literacy. Thus, a higher SES was associated with higher health literacy in the studied adolescents.

The biopsychological model (Table 4) proved to be significant *F* (7; 5628) = 169.281; *p* ≤ 0.001 and explained 17% of health literacy in the adolescents under study.

The variable associated with physical health (having or not having a diagnosed chronic disease) did not explain health literacy.

All the psychological variables (well-being, stress, and future expectations) proved to have statistically significant explanatory value for health literacy. Thus, well-being and future expectations positively predicted health literacy. In turn, stress negatively predicted health literacy, showing that higher well-being, less stress, and better future expectations were associated with higher health literacy in adolescents.

The model that contained lifestyle variables (Table 4) also proved to be significant *F* (14; 5621) = 50.340; *p* ≤ 0.001 explained 11% of health literacy and was composed of the following factors: body image, physical activity in the last 7 days, vegetable intake, up-to-date vaccinations, hours of sleep, screen time, consumption of tobacco, alcohol, and drugs, and involvement in violent behavior (as victim and aggressor).

From the analysis of the obtained model, satisfaction with body image, physical activity, vegetable intake, up-to-date vaccinations, and hours of sleep contributed positively to adolescents’ health literacy. On the other hand, screen time and violent behavior (as aggressor or victim) negatively predicted health literacy.

Therefore, having a more positive body image, practicing more physical activity, eating vegetables, having up-to-date vaccinations, having healthy sleep habits, spending less time exposed to screens, and not having engaged in violent behavior, were associated with higher health literacy in adolescents.

When analyzing the social factors related to health literacy (Table 4), the model was also significant *F* (8; 7346) = 146.573; *p* ≤ 0.001 and explained 14% of health literacy. All social variables (family support, the quality of relationships with teachers and peers, online contact with friends, and the use of social networks) contributed to adolescents’ health literacy. Family support and online contact with friends positively predicted health literacy. The relationship with teachers and peers (more value, worse relationship) and the use of social networks negatively predicted health literacy. Thus, better family support, better relationships with teachers and peers, more online contact with friends, and less use of social networks emerged as relevant variables for explaining adolescents’ health literacy.

Table 4 allows us to observe the health literacy model results with the environmental factors. This model was, once again, significant, *F* (6; 5561) = 15.567; *p* ≤ 0.001, explaining 1% of health literacy.

The following variables were considered to explain the contextual and environmental factors associated with adolescents’ health literacy: school pressure and perception of safety at school and in the community. From the analysis of this model, it was possible to verify that these variables explained health literacy: lower school pressure and a higher perception of safety at school predicted health literacy. An explanatory hypothesis for the size of the standardized coefficients (Table 3 and Table 4) involves the size of the sample, the large number of variables included in the analysis, and the dispersion of constructs and integrated dimensions, which, on the one hand, turns the variables into a more complete and ecological model but, on the other hand, makes it very diverse and potentially fragmented.

The analysis of all studied models (Table 5) showed us that a biopsychosocial approach is essential for understanding health literacy in Portuguese adolescents. Furthermore, the results showed that psychological factors had the most significant explanatory value for health literacy, followed by social factors and lifestyle. Sociodemographic and environmental factors revealed a more modest contribution in explaining health literacy in adolescents.

## 4. Discussion

The aim of the study was to gain an in-depth understanding of adolescents’ health literacy from a biopsychosocial and environmental perspective.

### 4.1. Main Findings of This Study

Health literacy is explained by factors related to physical, psychological, social, and environmental health. The factors with the greatest explanatory value were the psychological variables, followed by social and lifestyle-related variables. Sociodemographic and environmental factors had a more modest explanatory value.

The obtained results point to the complexity of adolescents’ health literacy, including their psychological and social characteristics, their access to information and knowledge, and even their ability to adopt healthier behaviors [4,29].

Sociodemographic control variables were considered, namely gender, age, and socioeconomic status. Only SES significantly explained health literacy, revealing that higher SES is associated with greater health literacy in adolescents [9,30].

On the other hand, having a diagnosed chronic illness did not reveal a statistically significant explanatory value for health literacy. This result was in line with that found by [31] in a systematic review in which chronic illness and physiological factors had no significant association with health literacy.

In the present study, psychological variables included well-being, stress management skills, and expectations for the future. All the variables proved to have statistically significant explanatory value for health literacy. Higher well-being and better expectations of the future were associated with higher health literacy among adolescents. More stress management difficulties were associated with lower health literacy among adolescents. Indicators of mental health and well-being were associated with higher health literacy. A study by [32] linked well-being and happiness with health literacy. Psychological health and psychological health literacy are associated with better mental health outcomes, better stress management skills and well-being [10,11,33].

The variables considered to characterize the lifestyle of the adolescents (body image, physical activity, vegetable intake, vaccinations, hours of sleep, screen time, consumption (tobacco, alcohol, and drugs) and involvement in situations of violence were also related to health literacy confirming the results obtained in other studies, about the relationship between lifestyle, health behaviors, and risk behaviors to adolescents’ health literacy. Eating, sleeping and physical exercise habits [13,15,17,21] are the factors that contribute most to adolescents’ health literacy.

Within the social variables under study (family support, quality of relationship with teachers and peers, online activities with friends, and use of social networks), family support, relationships with teachers, and relationships with peers were the factors that contributed most to adolescents’ health literacy. As with other factors related to health and well-being, social relationships, especially relationships with parents, are related to the quality of relationships with parents [34,35], teachers, and peers [14].

Finally, to understand the contextual and environmental factors associated with adolescents’ health literacy, we considered environmental variables (school pressure, perception of safety at school and in the community), evidencing that lower school pressure and a higher perception of safety at school were associated with higher health literacy.

The promotion of skills, knowledge, motivation, and opportunities is important to adopt healthy behaviors and to change risk behaviors. The school environment and the community in which adolescents live are prime locations for promoting health and health literacy. Health literacy can be integrated into curricula or be the focus of school projects involving students, families, teachers, and community partners (primary healthcare, municipalities, health, and social NGOs, etc.) [36,37].

### 4.2. Limitations of the Study

The assessment protocol used at HBSC/WHO is a global instrument for assessing the health of adolescents, and the measures used are generic in nature and not specific measurement instruments. However, the fact that it is a global measurement protocol allows us to have a very complete perspective of health literacy. On the other hand, the results, although representative of Portuguese students, cannot be generalized to other countries. Despite the limitations of the study, the obtained results are an important contribution and can be studied and replicated by other HBSC/WHO countries. The study includes several individual, interpersonal, and socioeconomic variables, but other relevant variables could be included, such as self-esteem, an in-depth study of family relationships, and socioeconomic characteristics, which in the literature appear to be fundamental to health literacy and access. Nevertheless, in the present study, these variables had a low, although significant, contribution. Aspects related to culture and specific regions of the country (north, south, inland, and coastal) may also help to explain adolescents’ health literacy. The development of quantitative studies including these variables and the development of qualitative research to deepen and identify the most effective strategies for promoting health literacy among adolescents and in their relevant contexts is of utmost importance

### 4.3. What This Study Adds

We conclude that a biopsychosocial approach helps to understand health literacy in adolescents. There is an association between socioeconomic status, psychological factors, lifestyle (especially health and protective behaviors), social relationships, and school environment that contribute to explaining health literacy in adolescents.

Psychological factors have the most explanatory value for health literacy, followed by social factors and lifestyle. Sociodemographic and environmental factors made a more modest contribution to the explanation of health literacy in adolescents.

Health literacy is developed throughout life considering experiences and opportunities. Family and school contexts can both contribute to health literacy and, consequently, to the promotion of mental health and well-being and the adoption of health behaviors and risk management. Health literacy promotion programs should follow common guidelines and be implemented in different contexts in a participatory process with continuous evaluation.

The health, education, and social sectors can use the information from this study to promote health literacy and health-promoting activities in a multidisciplinary, continuous, and consistent way, following the needs of adolescents in their development and, in the face of emerging external challenges, fostering the way that the family, health, education, and social sectors can contribute to building a friendly environment.

## Figures and Tables

**Table 1:** Characterization of participants.

Variables	% or *M* ± *SD*
Gender (*n* = 7355)	
Male	46.1 (3394)
Female	53.9% (3961)
Age (*n* = 7649)	15.05 ± 2.36 Min = 10.33; Max = 23.17
Socioeconomic status (SES) (*n* = 7649)	13.87 ± 2.19 Min = 7; Max = 19
Chronic disease (*n* = 7649)	
Yes	18.9 (1442)
No	81.1 (6207)
Well-being (*n* = 7649)	36.62 ± 7.14 Min = 12; Max = 50
Stress (*n* = 7649)	11.04 ± 3.06 Min = 4; Max = 20
Future expectations (*n* = 5881)	7.05 ± 2.08 Min = 0; Max = 10
Body image (*n* = 7649)	
I do not like my body	49.6 (3792)
I like my body	50.4 (3857)
Practice of physical activity in the last 7 days (*n* = 7649)	3.78 ± 2.04 Min = 0; Max = 7
Vegetable intake (*n* = 7649)	
Insufficient vegetable intake	65.1 (4983)
Healthy vegetable intake (every day)	34.9 (2666)
Updated vaccines (*n* = 7649)	
Not having up-to-date vaccinations	27.7 (2122)
Having up-to-date vaccinations	72.3 (5527)
Hours of sleep (*n* = 7649)	
Unhealthy sleeping habits	57.5 (4401)
Healthy sleep habits (9 h or more per night)	42.5 (3248)
Screen time (*n* = 5881)	22.99 ± 6.65 Min = 9; Max = 45
Lifetime cigarette consumption (*n* = 7649)	
Did not consume	84.8 (6490)
Consume	15.2 (1159)
Lifetime alcohol consumption (*n* = 7649)	
Did not consume	56.7 (4340)
Consume	43.3 (3309)
Drug use in the last month (*n* = 7649)	
Did not consume	95.8 (7331)
Consume	4.2 (318)
Violent behavior—Aggressor (*n* = 7649)	
Did not attack anyone	92.3 (7058)
Attacked someone	7.7 (591)
Violent behavior—Victim (*n* = 7649)	
Was not a victim	83.3 (6369)
Was a victim	16.7 (1280)
Family support (*n* = 7649)	22.67 ± 6.63 Min = 4; Max = 28
Relationship with teachers (*n* = 7649)	6.74 ± 2.54 Min = 3; Max = 15
Relationship with peers (*n* = 7649)	6.43 ± 2.54 Min = 3; Max = 15
Online contact with friends (*n* = 7649)	15.18 ± 4.41 Min = 4; Max = 24
Use of social networks (*n* = 7649)	15.08 ± 5.65 Min = 10; Max = 30
Pressure with school (*n* = 7649)	
Does not feel pressure	38.4 (2940)
Feels pressure	61.6 (4709)
School safety (*n* = 7649)	
Does not feel safe	5.2 (306)
Feels safe	94.8 (5575)
Safety in the area where you live (*n* = 7649)	
Does not feel safe	3.3 (194)
Feels safe	96.7 (5687)
Health literacy (*n* = 7649)	31.47 ± 5.08 Min = 10; Max = 40

**Table 2:** Measures and variables under study (measurements are part of the HBSC study protocol [23,24]).

Variables	Measure
Gender *	1—Male; 2—Female
Socioeconomic status (SES)	FAS Scale: Family Affluence Scale, six items reflecting the family’s material resources, such as owning a car or individual computer. The FAS score [26,27] was calculated for each adolescent based on the responses to these six items on a scale ranging from 0 to 13 points, with the highest values indicating better financial level.
Chronic disease *	1—Yes; 2—No
Well-being	Scale with 10 items with scores from 0 to 5. Results vary from 5 to 50. Higher values reveal a better perception of well-being.
Stress	Scale with four items with scores from 0 to 5. Results vary from 5 to 20. Higher values reveal greater stress.
Future expectations	Scale adapted from [28], composed of 11 steps, where the lowest step (0) corresponds to worst future expectations and the highest step (10) to better future expectations.
Body image *	1—I do not like my body; 2—I like my body
Practice of physical activity in the last 7 days	Scale from 0 to 7, where the lowest number (0) corresponds to 0 days of physical activity and the highest number (7) to 7 days of physical activity.
Vegetable intake *	1—Insufficient vegetable intake; 2—Healthy vegetable intake (every day)
Updated vaccines *	1—Not having up-to-date vaccinations; 2—Having up-to-date vaccinations
Hours of sleep *	1—Unhealthy sleeping habits; 2—Healthy sleep habits (9 h or more per night)
Screen Time	Scale with 10 items with scores from 1 to 5. Results vary from 5 to 50. Higher values reveal more screen time.
Lifetime cigarette consumption *	1—No consumption; 2—Consumption
Lifetime alcohol consumption *	1—No consumption; 2—Consumption
Drug use in the last month *	1—No consumption; 2—Consumption
Violent behavior—Aggressor *	1—Not aggressor; 2—Aggressor
Violent behavior—Victim *	1—Not a victim; 2—Victim
Family support	Scale with four items, on a 7-point Likert scale (1—very strongly disagree; 7—very strongly agree. Higher values reveal greater family support.
Relationship with teachers	Scale with three items on a five-point Likert scale (1—totally agree; 5—totally disagree). Higher values reveal a worse relationship with teachers.
Relationship with peers	Scale with three items on a five-point Likert scale (1—totally agree; 5—totally disagree). Higher values reveal a worse relationship with peers.
Online contact with friends	Scale with four items on a five-point Likert scale (1—totally agree;5—totally disagree). Higher values reveal more online contact with different types of friends.
Use of social networks	Scale with nine items about the use of different social networks in a dichotomic answer (No/Yes). Higher values reveal more diversity in using social networks.
Pressure with school *	1—Does not feel pressure; 2—Feels pressure
School safety *	1—Does not feel safe; 2—Feels safe
Safety in the area where you live *	1—Does not feel safe; 2—Feels safe
Health literacy	Scale with 10 items with scores from 0 to 5. Results vary from 5 to 50. Higher values reveal a better perception of health literacy.

* Variables with nominal measuring levels were coded as dummy variables.

**Table 3:** Linear regression of the variables under study for health literacy (Global Model).

	Nonstandardized Coefficient		Standardized Coefficient	*t*
	B	Standard Error	*β*	
Constant	17.66	1.41		12.59 ***
Gender	0.98	0.13	0.10	7.41 ***
Age	0.13	0.04	0.05	3.41 ***
Socioeconomic status (SES)	0.03	0.03	0.02	1.23
Chronic disease	−0.08	0.15	−0.01	−0.54
Well-being	1.49	0.14	0.21	10.94 ***
Stress	−0.08	0.03	−0.05	−3.05 **
Future expectations	0.25	0.03	0.11	7.72 ***
Body image	0.35	0.12	0.04	2.91 **
Practice of physical activity in the last 7 days	0.18	0.03	0.08	5.76 ***
Vegetable intake	0.96	0.13	0.09	7.57 ***
Updated vaccines	1.68	0.26	0.08	6.56 ***
Hours of sleep	0.32	0.12	0.03	2.75 **
Screen Time	−0.03	0.01	−0.03	−2.63 **
Lifetime cigarette consumption	0.24	0.18	0.02	1.35
Lifetime alcohol consumption	0.24	0.13	0.02	1.78
Drug use in the last month	−0.61	0.29	−0.03	−2.07 *
Violent behavior—Aggressor	−0.76	0.24	−0.04	−3.16 **
Violent behavior—Victim	−0.28	0.18	−0.02	−1.54
Family support	0.05	0.01	0.07	4.64 ***
Relationship with teachers	−0.22	0.08	−0.04	−2.58 *
Relationship with peers	−0.06	0.03	−0.03	−2.37 *
Online contact with friends	0.07	0.02	0.06	4.82 ***
Use of social networks	−0.04	0.01	−0.04	−3.41 ***
School pressure	0.39	0.14	0.04	2.77 **
School safety perception	−0.84	0.28	−0.04	−2.99 **
Community safety perception	−0.05	0.33	−0.00	−0.15

The variables were entered using the “enter” mode. * *p* ≤ 0.05; ** *p* ≤ 0.01; *** *p* ≤ 0.001.

**Table 4:** Linear regression models biopsychosocial and environmental variables for health literacy.

	Nonstandardized Coefficient		Standardized Coefficient	*t*
	B	Standard Error	*β*	
*Sociodemographic variables for the health literacy model*				
Constant	27.33	0.79		34.69 ***
Gender	−0.02	0.13	−0.00	−0.13
Age	0.05	0.04	0.02	1.23
Socioeconomic status (SES)	0.24	0.03	0.11	8.12 ***
*Biopsychological variables for the health literacy model*				
Constant	16.44	1.06		15.48 ***
Gender	1.19	0.13	0.12	9.38 ***
Age	0.17	0.03	0.06	5.06 ***
Socioeconomic status (SES)	0.11	0.03	0.05	4.10 ***
Chronic disease	−0.10	0.15	−001	−0.63
Well-being	2.17	0.12	0.31	17.67 ***
Stress	−0.10	0.03	−0.06	−3.67 ***
Future expectations	0.33	0.03	0.14	9.98 ***
*Lifestyle variables for the health literacy model*				
Constant	20.69	0.90		23.13 ***
Gender	0.22	0.17	0.02	1.70
Age	0.10	0.04	0.04	2.50 **
Socioeconomic status (SES)	0.13	0.03	0.06	4.30 ***
Body image	1.08	0.13	0.11	8.55 ***
Practice of physical activity in the last 7 days	0.36	0.03	0.15	10.98 ***
Vegetable intake	1.34	0.13	0.13	10.06 ***
Updated vaccines	2.25	0.27	0.11	8.32 ***
Hours of sleep	0.70	0.13	0.07	5.62 ***
Screen time	−0.02	0.01	−0.03	−2.44 *
Lifetime cigarette consumption	−0.17	0.19	−0.01	−0.93
Lifetime alcohol consumption	−0.03	0.14	−0.00	−0.18
Drug use in the last month	0.87	0.31	−0.04	−2.79
Violent behavior—Aggressor	−1.13	0.26	−0.06	−4.42 ***
Violent behavior—Victim	−0.95	0.19	−0.07	−4.98 ***
*Social variables for the health literacy model*				
Constant	25.96	0.66		39.41 ***
Gender	0.57	0.11	0.06	5.13 ***
Age	1.04	0.02	0.02	1.60
Socioeconomic status (SES)	0.17	0.03	0.07	6.68 ***
Family support	0.16	0.01	0.21	18.13 ***
Relationship with teachers	−0.27	0.03	−0.13	−10.58 ***
Relationship with peers	−0.15	0.03	−0.08	−6.11 ***
Online contact with friends	0.11	0.01	0.10	8.79 ***
Use of social networks	−0.06	0.01	−0.07	−6.18 ***
*Environment for the health literacy model*				
Constant	24.02	1.21		19.87 ***
Gender	0.16	0.14	0.12	1.18
Age	0.07	0.04	0.02	1.79
Socioeconomic status (SES)	0.23	0.03	0.10	7.73 ***
School pressure	−0.54	0.15	−0.05	−3.60 ***
School safety perception	1.10	0.31	0.05	3.56 ***
Community safety perception	0.83	0.37	0.03	2.27 *

The variables were entered using the “enter” mode. * *p* ≤ 0.05; ** *p* ≤ 0.01; *** *p* ≤ 0.001.

**Table 5:** Analysis of linear regression models for health literacy.

	*R* ^2^	*F*	*gl*	*p*
Global model	0.22	61.121	26	≤0.001
Sociodemographic model	0.01	21.984	3	≤0.001
Biopsychological model	0.17	169.281	7	≤0.001
Lifestyle model	0.11	50.340	14	≤0.001
Social model	0.14	146.573	8	≤0.001
Environmental model	0.01	15.567	6	≤0.001

## Data Availability

Data cannot be publicly shared.
